# Object Occlusion Detection Using Automatic Camera Calibration for a Wide-Area Video Surveillance System

**DOI:** 10.3390/s16070982

**Published:** 2016-06-25

**Authors:** Jaehoon Jung, Inhye Yoon, Joonki Paik

**Affiliations:** 1 Department of Image, Chung-Ang University, 84 Heukseok-ro, Dongjak-gu, Seoul 06974, Korea; gjslkjs@gmail.com (J.J.); inhyey@gmail.com (I.Y.); 2 ADAS Camera Team, LG Electronics, 322 Gyeongmyeong-daero, Seo-gu, Incheon 22744, Korea

**Keywords:** occlusion detection, automatic camera calibration, depth estimation, moving object detection, video surveillance system

## Abstract

This paper presents an object occlusion detection algorithm using object depth information that is estimated by automatic camera calibration. The object occlusion problem is a major factor to degrade the performance of object tracking and recognition. To detect an object occlusion, the proposed algorithm consists of three steps: (i) automatic camera calibration using both moving objects and a background structure; (ii) object depth estimation; and (iii) detection of occluded regions. The proposed algorithm estimates the depth of the object without extra sensors but with a generic red, green and blue (RGB) camera. As a result, the proposed algorithm can be applied to improve the performance of object tracking and object recognition algorithms for video surveillance systems.

## 1. Introduction

Recently, the demand for object tracking and recognition algorithms is increasing due to video surveillance. An object occlusion is a major factor for the performance degradation of a video surveillance system. For this reason, various object occlusion detection and handling methods were studied.

Mei et al. proposed an object tracking with consideration of occlusion that is detected using the occlusion map [1]. Since this method uses a target template to obtain the occlusion map, it is difficult to detect the object occlusion when the target template is unavailable. Zitnick et al. generated a depth map using a stereo camera and detected object occlusion regions [2]. However, this method needs two cameras for stereo matching to generate the depth map. Sun et al. proposed an optimization approach using the visibility constraint for the stereo matching and then generated the depth map by minimizing the energy function [3]. Since a stereo camera-based occlusion detection method needs an additional camera, it is not easy to implement in an already installed wide-area surveillance system.

To solve this problem, single camera based depth map estimation methods were proposed. Matyunin et al. estimated the depth using an infrared sensor [4]. However, this method cannot work in the outdoor scene since an infrared sensor is interrupted by sunlight. Im et al. proposed a single red, green, and blue (RGB) camera-based object depth estimation method using multiple color-filter apertures (MCA) [5]. However, this method needs a special aperture for the object depth estimation, and produces color distortion at boundary of the out-focused objects. Zonglei et al. used a patterned box for semi-automatic camera calibration [6]. Lin et al. estimated vanishing points using traffic lanes, and estimated the distance of a frontal vehicle using a single RGB camera for a collision warning system [7]. Since this method uses the traffic lane for vanishing point estimation, distance estimation is impossible when an input image does not contain a traffic lane. Song et al. detected features from a moving object, and automatically calibrated the camera [8]. However, Song’s method cannot avoid the camera calibration error when feature points change while the object is moving.

To solve these problems, the proposed method first performs automatic camera calibration using both moving objects and background structures to estimate camera parameters. Given the camera parameters, the proposed algorithm estimates the object depth with regard to a reference plane, and then detects the object occlusion. To estimate vanishing points and lines, the proposed algorithm detects parallel lines in the input image. Bo et al. detected straight lines from background structures using the one-dimensional (1D) Hough transform for automatic camera calibration [9]. Since this method uses only a single image, it is impossible to automatically calibrate the camera when the background does not have line components. Moreover, accuracy of the camera calibration is degraded by with non-parallel lines. Lv et al. detected human foot and head points for automatic camera calibration [10]. However, if the estimated foot and head points are not sufficiently accurate or if the object motion is linear, the camera calibration is impossible. Moreover, accuracy of the camera calibration result depends on the object detection results. To solve these problems, the proposed algorithm combines the background structure lines with human foot and head information to estimate vanishing points and lines.

This paper is organized as follows: Section 2 describes background theory of the camera geometry, and Section 3 presents the proposed object occlusion detection algorithm. Experimental results of the proposed algorithm are shown in Section 4, and Section 5 concludes the paper.

## 2. Theoretical Background of Camera Geometry

Estimation of the depth needs a 3D space information. To obtain the projective relationship between the 2D image and 3D space information, a camera geometry is used with camera parameter that describe camera sensor, lens, optical axis, and a position of the camera in the world coordinate. In the pin-hole camera model [11], a point in the 3D space is projected onto a point in the 2D image as

(1) $$s\left[\begin{array}{c} x\\ y\\ 1 \end{array}\right]=\left[\begin{array}{ccc} \begin{array}{c} f_{x}\\ 0\\ 0 \end{array} & \begin{array}{c} skew\\ f_{y}\\ 0 \end{array} & \begin{array}{c} p_{x}\\ p_{y}\\ a \end{array} \end{array}\right]\left[\begin{array}{cccc} \begin{array}{c} r_{11}\\ r_{21}\\ r_{31} \end{array} & \begin{array}{c} r_{12}\\ r_{22}\\ r_{32} \end{array} & \begin{array}{c} r_{13}\\ r_{23}\\ r_{33} \end{array} & \begin{array}{c} t_{1}\\ t_{2}\\ t_{3} \end{array} \end{array}\right]\left[\begin{array}{c} X\\ Y\\ Z\\ 1 \end{array}\right]=A\left[R|t\right]\left[\begin{array}{c} X\\ Y\\ Z\\ 1 \end{array}\right]$$

where *s* represents the scale, ${\left[\begin{array}{ccc} x & y & 1 \end{array}\right]}^{T}$ a point in the 2D image, matrix A consists of intrinsic camera parameters, $f_{x}$ and $f_{y}$ focal length in the x- and y-axis focal length, respectively, skew the skewness, *a* the aspect ratio, camera rotation matrix R consists of camera rotation parameters $r_{ij}$, ${\left[\begin{array}{ccc} t_{1} & t_{2} & t_{3} \end{array}\right]}^{T}$ the camera translation vector, and ${\left[\begin{array}{cccc} X & Y & Z & 1 \end{array}\right]}^{T}$ a point in the 3D space.

To simplify the description without loss of generality, we assume that the focal lengths $f_{x}$ and $f_{y}$ are equivalent, the principal point is at the image center, the skewness is equal to zero, and the aspect ratio is equal to 1. In the same manner, we also assume that the camera rotation angle with regard to the Z-axis is equal to zero, and the camera translation with regard to both X- and Y-axis is equal to zero to calculate the extrinsic matrix [R|t] as

(2) $$\left[R|t\right]=\left[R_{Z}\left(\rho \right)R_{X}\left(\theta \right)T\left(0,0,h_{c}\right)\right]$$

where $R_{Z}$ represents the rotation matrix with regard to the Z-axis, $R_{X}$ the rotation matrix with regard to the X-axis, *T* the transformation for a translation, $h_{c}$ the camera height.

**Figure 1:**
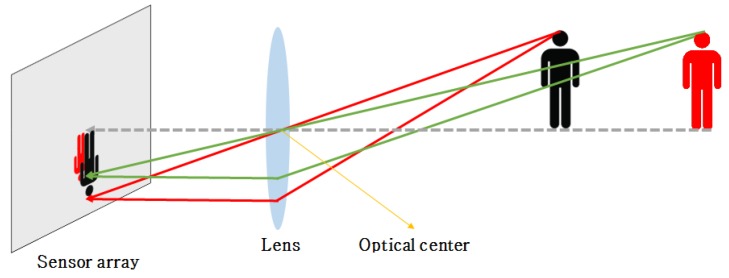
Projective model of the camera.

The 2D image is generated by the light that is reflected by an object and than arrives at the camera sensor. In this process, a single object is projected onto the 2D image plane with different sizes according to the distance from the camera as shown in Figure 1. For this reason, parallel lines in the 3D space are projected on the 2D image plane as non-parallel lines depending on the depth. Using the apparent non-parallel lines in the image, the vanishing points can be estimated as a intersected points of those lines. Since the projective camera transformation model projects a point in the 3D space onto the camera sensor, the camera parameters can be estimated using vanishing points.

The point in the 3D space is projected onto a camera sensor corresponding to a point in the 2D image using a projective transform. However, a point in the 2D image cannot be inversely projected onto a unique point in the 3D space since the camera projection transform is not a one-to-one function. On the other hand, if there is a reference plane, a point in the 2D image can be inversely projected onto a point on the reference plane that is defined in the 3D space. As a result, the proposed algorithm estimates the object depth using the 2D image based on a pre-specified reference plane.

## 3. Automatic Calibration-Based Occlusion Detection

**Figure 2:**
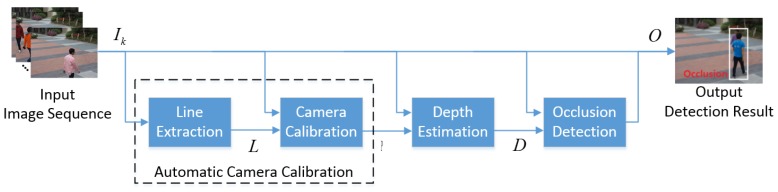
Block diagram of the proposed occlusion detection algorithm.

The proposed object occlusion detection algorithm consists of three steps: (i) automatic camera calibration; (ii) object depth estimation; and (iii) occlusion detection. Figure 2 shows the block diagram of the proposed algorithm, where $I_{k}$ represents the *k*-th input frame, *L* represents the extracted lines, *P* represents the projective matrix, *D* represents the object depth information, and *O* represents the detected region of an occluded object.

### 3.1. Automatic Camera Calibration

The proposed algorithm estimates the camera parameters for the object depth estimation followed by object occlusion detection. Since semi-automatic camera calibration is the simplest way to estimate parameters using a synthetic calibration pattern [6], its performance is highly dependent on the experience of a user. To solve this problem, the proposed algorithm uses an automatic camera calibration method that extracts lines from the image, and then estimates vanishing points and lines [12].

To detect human foot and head points, the proposed algorithm detects the foreground by modeling the background using the Gaussian mixture model (GMM) [13]. The object region is then detected by labeling a sufficiently large object. Given an object region, the vertically highest point is determined as the head point. On the other hand, the average of the bottom 20 percent points is determined as the foot point.

A pair of parallel lines that connect head points and foot points are used to detect vanishing points and lines. Since the lines connecting head points and foot points are non-parallel when the height of an object changes while walking, the proposed algorithm detects the uniform height of the object only when pedestrian’s legs are crossing as

(3) $$\frac{1}{n}\sum_{i=1}^{n}{\left(p_{i}-p_{f}\right)}^{2}<T_{C}$$

where $p_{i}$ represents the candidate foot points, *n* the number of candidate foot points, $p_{f}$ the detected foot point, and $T_{C}$ the threshold value.

**Figure 3:**
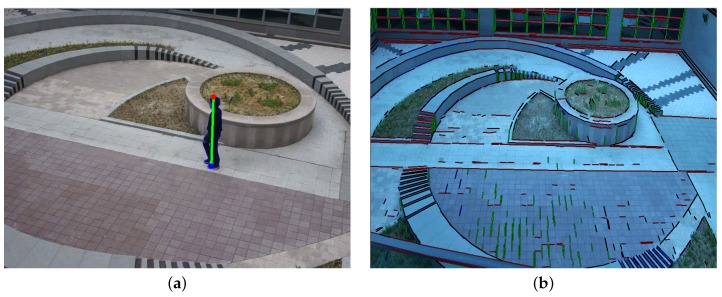
Results of the line detection: (**a**) foot-to-head line and (**b**) background structure lines.

To combine object foot and head information with the background structure lines, the proposed algorithm detects edges that are used to detect vanishing points and lines [14]. The detected foot and head points and background structure lines are shown in Figure 3.

**Figure 4:**
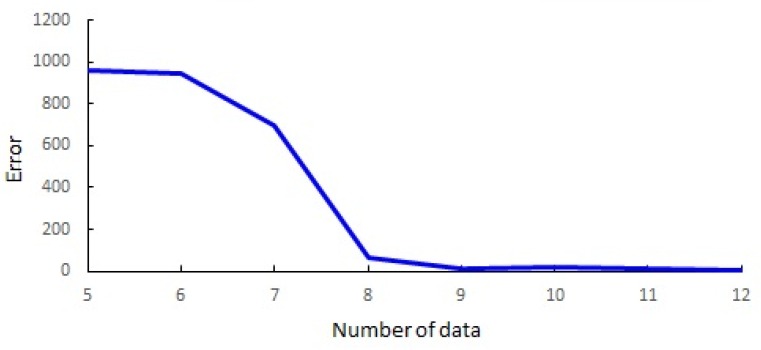
Focal length estimation error depending on the number of foot-head data sets with 30% outliers.

The vanishing points and lines are estimated using the detected foot-to-head line and background structure lines. For the robust vanishing points and lines estimation, a sufficient number of foot and head points are required. For that reason, the proposed algorithm estimates the vanishing points and lines depending on the number of the human foot and head points according to the following three cases:

Theoretically, homography estimation for camera calibration requires four 2D coordinates that can solve eight linear equations. However, a practical random sample consensus (RANSAC) based robust camera calibration needs at least eight points such that the calibration error is minimized as shown in Figure 4.

Case 1. If the number of detected foot and head point sets is less than *N*, the vanishing points and lines are estimated using background structure lines. More specifically, three vanishing points are selected from background lines intersecting points using the RANSAC algorithm. Among three vanishing points, the lowest one is determined as the vertical vanishing point. The line connecting the remaining two vanishing point is determined as the horizontal vanishing line.

**Figure 5:**
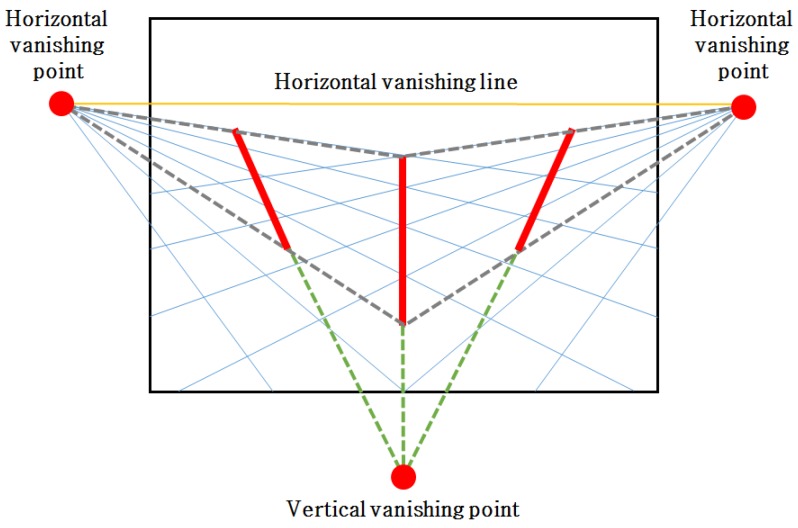
Definition of vanishing points and the horizontal vanishing line.

Case 2. If the number of detected foot and head sets is more than *N* but the object motion is linear, the vertical vanishing point can be estimated only using the object foot and head points. The vertical vanishing point is determined at the intersected point of foot-to-head lines as shown in Figure 5. However, if the object moves linearly, estimation of a horizontal vanishing line is impossible since only one horizontal vanishing point is estimated. In this case, the vanishing line is estimated using background structure lines.

Case 3. If the number of detected foot and head sets is more than *N* and object motion is not linear, vanishing points and lines can be estimated using foot and head points. A foot-to-foot line that connects two foot points and the corresponding head-to-head line that connects two head points are used to estimate the horizontal vanishing points. As a result, the horizontal vanishing line can be estimated using the two horizontal vanishing points as shown in Figure 5.

Camera parameters are calculated using the estimated vertical vanishing point and the horizontal vanishing line as [15]

(4) $$\begin{array}{c} f=\sqrt{\left(a_{3}/a_{2}-p_{y}\right)\left(v_{y}-p_{y}\right)}\\ \rho =\mathrm{atan}(-v_{x}/v_{y})\\ \theta =\mathrm{atan}(-\sqrt{v_{x}^{2}+v_{y}^{2}}/f)\\ h_{c}=h_{o}/\left(1-\frac{d\left(o_{h},v_{l}\right)∥o_{f}-v∥}{d\left(o_{f},v_{l}\right)∥o_{h}-v∥}\right) \end{array}$$

where *f* represents the focal length, *ρ* the roll angle, *θ* the tilt angle, $h_{c}$ the camera height, $v_{l}$ the horizontal vanishing line $a_{1}x+a_{2}y+a_{3}=0$, $v={\left[\begin{array}{cc} v_{x} & v_{y} \end{array}\right]}^{T}$ the vertical vanishing point, $h_{o}$ the object height, $o_{f}$ the object foot point, $o_{h}$ the object head point, and d(A,B) the distance between a point *A* and a line *B*.

### 3.2. Object Depth Estimation and Occluded Region Detection

The proposed algorithm uses object depth information to detect an occluded region. To estimate the depth of an object, the 2D image coordinate is projected onto the reference plane in the 3D space using a projective matrix. Since the object foot points should be on the ground plane, the proposed algorithm uses the ground plane as the reference plane, which means that the ground plane is considered as the XY plane because the camera height is calculated as the distance between the ground plane and the camera. Using an object foot point in the 2D image, the object foot point on the ground plane in the 3D space can be calculated. To detect the foot point in the 3D space, a foot point in the 2D image is inversely projected onto the 3D space using a projective matrix as

(5) $$X={\left(P^{T}P\right)}^{-1}P^{T}x_{f}$$

where $x_{f}$ represents the foot point in the 2D image, matrix P the projective matrix, and X the inversely projected coordinate of $x_{f}$. Inversely projected coordinate X is normalized by the Z-axis value to detect the foot point in the 3D space as

(6) $$X_{f}=\frac{X}{Z}$$

where *Z* represents the Z-axis value of X, and $X_{f}$ the foot point on the ground plane in the 3D space.

**Figure 6:**
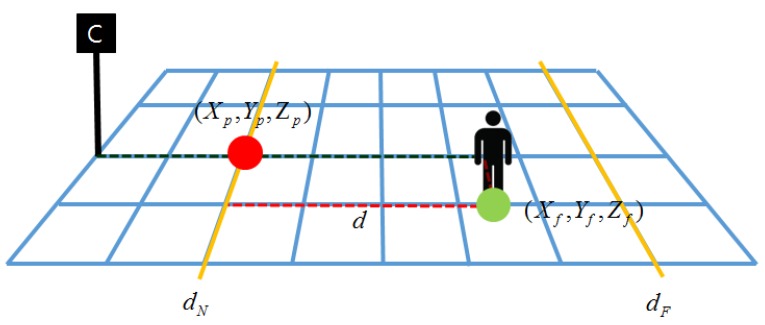
The proposed object depth estimation model.

An object depth is estimated by computing the distance between the object and camera. However, the foot point appears in the finite position in the input image. For this reason, the proposed algorithm uses the nearest foot point as a pivot point for the object depth estimation. The estimated depth is then normalized using the farthest distance. If an object is far enough from the camera, depth of the object foot point is assumed to be the Y-axis coordinate since the camera pan angle is equal to zero and the pivot point is on the ground plane. If the object depth is equal to the object foot point depth, the object depth is calculated as

(7) $$d=\frac{\left|Y_{f}-Y_{p}\right|}{d_{F}}$$

where *d* represents the object depth, $Y_{p}$ the Y-axis value of the pivot point, $Y_{f}$ the object foot point, and $d_{F}$ the farthest distance. Figure 6 shows the proposed object depth estimation model, where $d_{N}$ represents the nearest distance, $\left(X_{p},Y_{p},Z_{p}\right)$ the pivot point, and $\left(X_{f},Y_{f},Z_{f}\right)$ the object foot point.

The proposed algorithm detects the object occlusion using the estimated object depth. The depth of the same object in adjacent frames slowly changes. On the other hand, if the object is occluded, the estimated depth of the object rapidly changes. Based on the observation, object occlusion is detected as

(8) $$O=\left\{\begin{array}{cc} \mathrm{true}, & \mathrm{if}\left|d_{t-1}-d_{t}\right|\geq T_{O}\\ \mathrm{false}, & \mathrm{otherwise} \end{array}\right.$$

where *O* represents the object occlusion detection result, $d_{t}$ the depth of the object at time *t*, and $T_{O}$ the threshold value for the object occlusion detection. We can only estimate depth from standing human objects whose feet lie on the reference plane assuming that each object has a uniform depth.

## 4. Experimental Results

This section shows the results of the proposed automatic camera calibration and object occlusion detection algorithms. For the experiment, test video sequences of resolution 1280 × 720 were acquired using a camera installed at 2.2 to 7.2 m high. In addition to the in-house test sets, Vision and Autonomous System Center’s (VASC) stereo dataset is also used to compare the performance of the proposed method with existing stereo matching-based methods [16].

**Figure 7:**
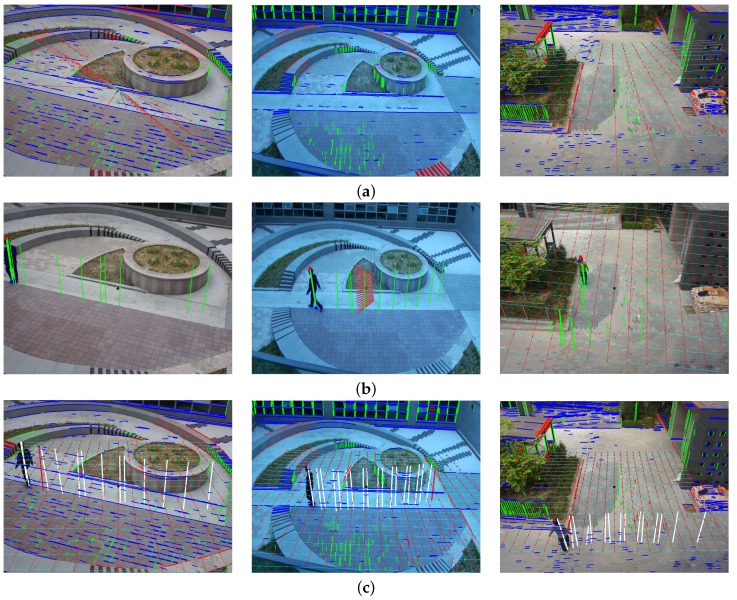
Results of three different method for camera calibration: (**a**) background structure-based method; (**b**) moving object-based method; and (**c**) the proposed camera calibration method.

Figure 7 shows the result of three different methods for automatic camera calibration. A ground plane is drawn on the image using grid lines with a 0.5 m interval to show the accuracy of the camera calibration. The background structure-based method makes a poor calibration result because of insufficient, non-parallel line segments and random textures of natural objects as shown in Figure 7a. The moving object-based method degrades the calibration performance because of the incompletely detected moving object and only linear motion of the object as shown in Figure 7b. On the other hand, the proposed method significantly improves the accuracy of camera calibration because it uses neither incomplete background structures nor multiple object positions in the same line as shown in Figure 7c.

**Figure 8:**
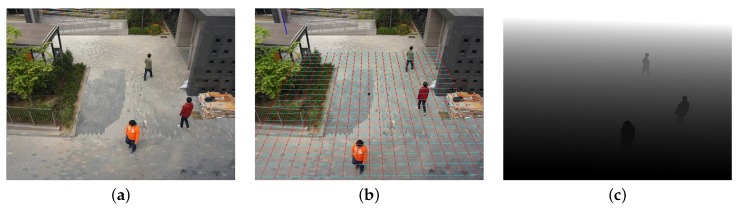
The results of the proposed object depth estimation: (**a**) input image; (**b**) calibration result in the form of the superimposed grid representing the ground plane; and (**c**) the depth estimation result.

Figure 8 shows the result of object depth estimation using the proposed method. Figure 8b shows the calibration result with the superimposed ground plane. The estimated depths of moving objects are shown in Figure 8c.

**Figure 9:**
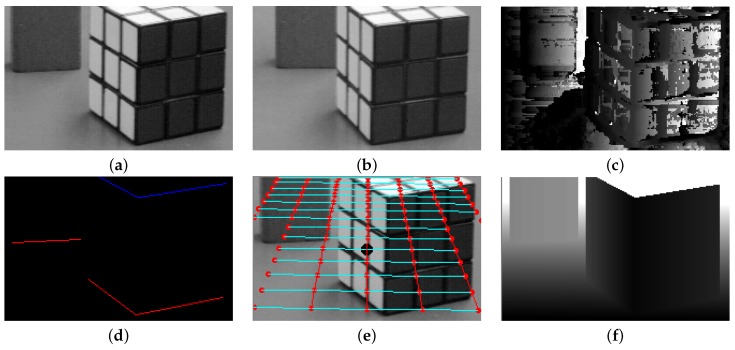
Comparison of depth estimation results using the stereo-based and the proposed methods: (**a**) the left stereo image; (**b**) the right stereo image; (**c**) estimated depth map using the stereo matching-based method; (**d**) guide lines of the left image; (**e**) estimated ground plane of the left image; and (**f**) estimated depth map of the left image using the proposed method.

Figure 9 compares depth estimation results using the stereo matching-based and the proposed methods. Figure 9a,b respectively show the left and right images of the “Toy” in the VASC stereo dataset. Figure 9c shows the stereo matching-based depth estimation result. Figure 9d shows guidelines to detect an region, where red lines represent the objects bottom boundary and blue lines the object’s top boundary. Figure 9e shows the superimposed grid of the reference plane that is the camera calibration result. The calibration result using the proposed object depth estimation method is shown in Figure 9f. Although the stereo matching-based method generated many holes in textureless regions without features, the proposed method successfully estimated the continuous depth map.

**Figure 10:**
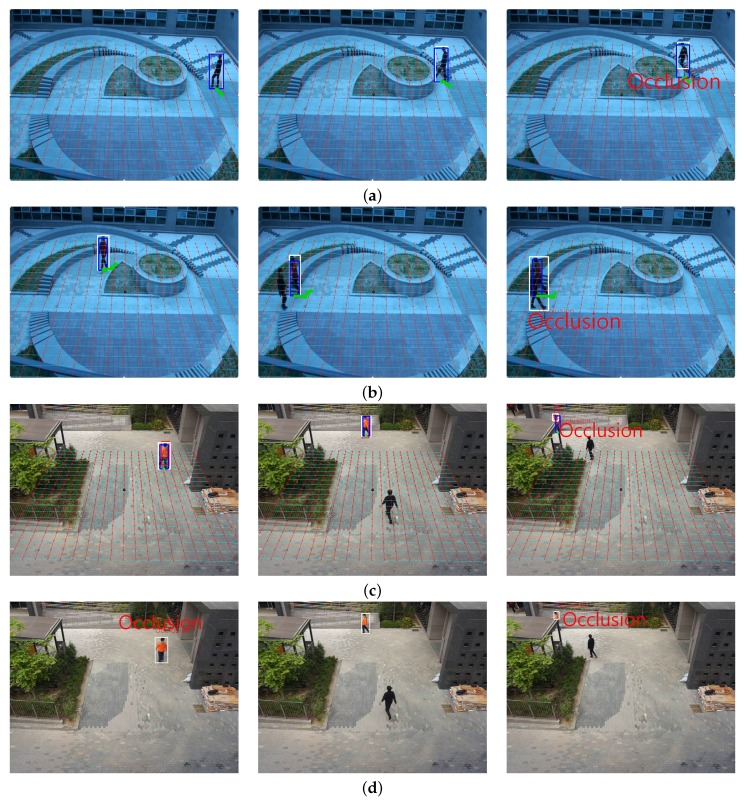
Results of occlusion detection three selected frames in each video: (**a**) occlusion by background; (**b**) occlusion by another object; (**c**) result of occlusion detection detection in another video and (**d**) result occlusion detection without depth information.

Figure 10 shows the detection result of an occluded object using the proposed occlusion detection algorithm with the threshold distance of 1.0 m. Figure 10a shows the detection result of the occluded object by a background structure. Figure 10b shows the detection result of the occluded object by another object, and Figure 10c shows the detection result in a different test video. The proposed method can successively detect occlusion in various test videos. As shown in Figure 10d detection of the y-axis value of an object foot position may results in erroneous detection of occlusion. However, the proposed method can correctly detect occlusion in the scene-invariant manner since it uses the depth in formation in the 3D space.

## 5. Conclusions

In this paper, we presented a fully automatic object occlusion detection method by estimating the object depth from a single uncalibrated camera. The proposed algorithm can robustly calibrate a camera by combining the background structure line components and moving object information. In addition, object depth is estimated using a single RGB camera. As a result, the object occlusion is successfully detected by analyzing the object depth information. The proposed method can be applied to object detection and tracking in a multiple-view surveillance system.

The fundamental assumption of the proposed occlusion detection algorithm is that there is a single, flat ground on which all objects move around. If the ground is not flat or slanted, the estimated depth becomes inaccurate, and as a result, object detection may fail. In that case, the nonflat ground can be approximated by piece-wise flat one, and the slanting ground can be taken care of in the calibration process. In spite of the restrictions, the proposed method is suitable for a wide range of surveillance applications, such as multiple camera video tracking with object handover and normalized metadata generation-based video indexing and retrieval because of its economical implementation.
